# Hepatitis B Virus: Infection, liver disease, carcinogen or syndemic threat? Remodelling the clinical and public health response

**DOI:** 10.1371/journal.pgph.0001359

**Published:** 2022-12-02

**Authors:** Philippa C. Matthews, Tongai Maponga, Indrajit Ghosh, Maud Lemoine, Ponsiano Ocama, Ibrahim Abubakar, Alistair Story, Stuart Flanagan

**Affiliations:** 1 The Francis Crick Institute, London, United Kingdom; 2 Division of Infection and Immunity, University College London, London, United Kingdom; 3 Department of Infectious Diseases, University College London Hospitals, London, United Kingdom; 4 Division of Virology, Tygerberg Hospital, University of Stellenbosch, Stellenbosch, South Africa; 5 Mortimer Market Centre, Central North West London NHS Foundation Trust, London, United Kingdom; 6 Find & Treat, Inclusion Health, University College London, London, United Kingdom; 7 Department of Metabolism, Digestion and Reproduction, Division of Digestive Diseases, Section of Hepatology, Imperial College London, London, United Kingdom; 8 Makerere University College of Health Sciences, Kampala, Uganda; 9 Faculty of Population Health Sciences, University College London, Mortimer Market Centre, London, United Kingdom; Stellenbosch University, SOUTH AFRICA

## Background

Hepatitis B virus (HBV) is a blood-borne virus that establishes chronic liver infection, associated with potential complications of cirrhosis and/or hepatocellular carcinoma (HCC). As such, it poses a major threat to global public health, accounting for 300 million chronic infections, and more than 500,000 deaths each year [1]. It can also be regarded as a ‘syndemic’ challenge—namely, as one component of multiple, complex interacting health and social needs.

The global prevalence of HBV infection is estimated at 4%, but the burden of morbidity and mortality is focused in the WHO Africa and Western Pacific regions, where the population prevalence is 6–7% [2], and healthcare delivery is poorly resourced. Outside these settings, HBV disproportionately affects marginalised groups who may lack reliable access to healthcare services (including refugees and migrants, the LQBTQ+ community, people experiencing homelessness, injecting drug users, incarcerated people, and sex workers).

Under the umbrella of international Sustainable Development Goals, specific elimination targets have been set for viral hepatitis for the year 2030 [3]. Clinical guidelines provide recommendations for HBV prevention, screening, surveillance and treatment (e.g. [4–6]). Preventive measures mainly focus on immunisation. Vaccination is combined with maternal screening and antiviral prophylaxis, with or without Hepatitis B immunoglobulin (HBIg), as a package of measures to reduce mother-to-child transmission. Diagnosis requires access to screening for hepatitis B surface antigen (HBsAg), ideally supported by testing for HBeAg and HBV viral load (VL), while surveillance and risk-stratification demands regular follow-up with laboratory assays (to monitor VL and liver health) alongside imaging with elastography and/or ultrasound.

Treatment for those meeting eligibility criteria is typically with a long-term oral nucleos(t)ide analogue agent (e.g. tenofovir, entecavir). However, a minority of those diagnosed with chronic infection qualify for treatment, among whom only a further minority can reliably and affordably access monitoring and medication. Furthermore, clinical guidelines are not uniform, do not cover all clinical scenarios (such that a substantial proportion of patients fall into ‘grey zones’) and are difficult or impossible to apply in some settings due to resource constraints [7]. Substantial health inequities represent barriers to the global attainment of elimination targets.

**Fig 1:**
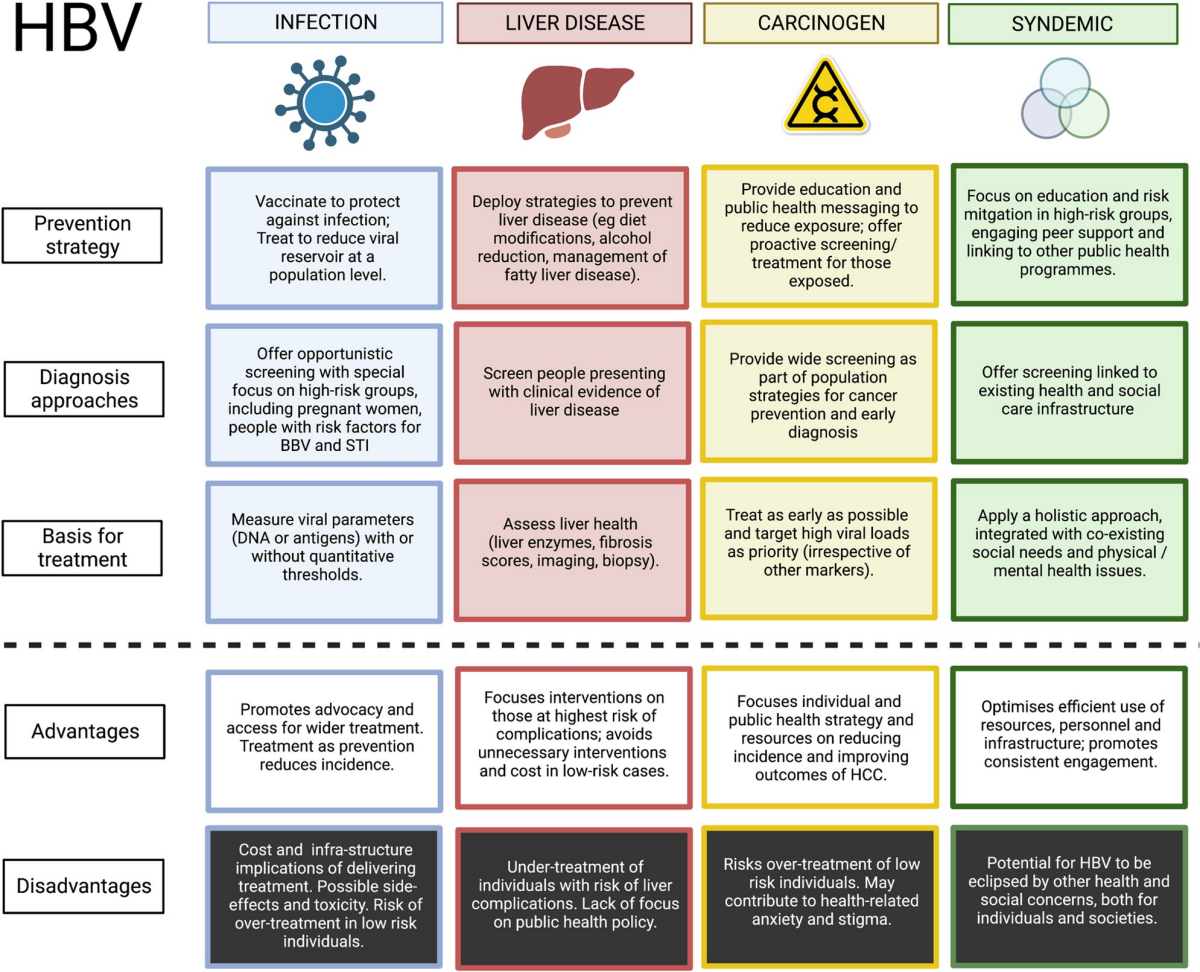
Summary of clinical and public health approaches to HBV that are driven by viewing it as an infection, liver disease, carcinogen or syndemic challenge. Figure created using BioRender with a licence to publish.

By viewing HBV primarily as a cause of liver disease, an infectious agent, a carcinogen, or as a syndemic challenge, clinical and public health approaches can be distinctly shaped and refined (Fig 1). We discuss the ramifications of designing HBV strategies specific to each of these ‘lenses’, and highlight the urgent need to align an integrated response.

## HBV as a liver disease

Management of HBV as a liver disease is underpinned by laboratory tests, radiological investigation, and/or histopathological assessment. Guidelines apply an algorithmic approach to these results to determine end-organ disease, alongside risk factors including age, sex, ethnic origin, and family history of liver cancer [4–6]. Typically, if objective measurements of liver health are normal, HBV is not treated, irrespective of virological markers or risk factors. This approach is exemplified by the management paradigm for children and teenagers in which antiviral treatment is not routinely offered as there is typically no laboratory or imaging evidence of liver disease, despite potentially high VL and HBeAg-positive status (a phenotype previously described as ‘immune tolerant’).

This stratification of therapy aims to target those at highest short-term risk of liver disease, and avoids unnecessary treatment of individuals with the lowest chances of developing long-term complications. However, the approach requires significant clinical infrastructure to maintain regular surveillance, and is associated with unchecked viral replication (with the risk of viral integration at high VL, and liver disease evolution even at low VL [8]), while sustained viraemia is a reservoir for ongoing community transmission.

## HBV as an infection

Viewing HBV primarily as an infectious agent builds on established approaches for other chronic blood-borne viruses, such as HCV and HIV, as well as containment approaches to other viruses that threaten population health. Suppressing or clearing HBV or HCV lowers the long-term risks of cirrhosis and liver cancer, liver enzymes and fibrosis scores can improve over time [9], and there is a benefit to quality of life [10]. Approaches to BBV prevention are unified by common principles, as reductions in viraemia translate into a lowered transmission risk. For HIV this is headlined by the public health message ‘undetectable = untransmittable’ (U = U) based on a reduction in new infections in treated populations [11]. During the SARS-CoV-2 pandemic, public health measures implemented due to the risk of severe disease (albeit occurring in a minority), aimed at strict containment, irrespective of the specific clinical phenotype in infected individuals.

HBV vaccination has reduced HBV and HCC incidence, but is not sufficient to achieve elimination targets due to the large established population reservoir of HBV infection, gaps in vaccine-mediated immunity, and practical challenges in delivering a universal three-dose vaccine regimen starting at birth. While prevention for HBV is ideally tackled by population immunisation, there is an argument for tackling established infection by treating early and aiming for universal virologic suppression [7].

Quantification of VL and viral antigens (HBsAg and HBeAg) and DNA provide a proxy for viral replication, and are universally incorporated into treatment algorithms. However, considering HBV as a high-risk agent of infection would uncouple treatment from specific quantitation of any laboratory markers, thus minimising both the potential for long-term liver disease (in individuals), and reducing onward transmission (in populations). For those with established infection, this perspective supports mandating universal access to first-line treatment, which would simplify and unify clinical algorithms, reduce the clinical infrastructure required for treatment stratification, and redress inequities. The approach has to account for the risks and costs of long-term therapy, even when the individual risk of complications or transmission are small.

## HBV as a carcinogen

The lifetime risk of HCC in chronic HBV infection has been estimated as 27% in males and 8% in females, and broadly increases with VL, ranging from 108 per 100,000 person-years for an HBV DNA level of <300 copies/mL to >1000 per 100,000 person-years for VL 1 million copies/mL [12]. Cancer incidence is higher in the context of cirrhosis, with a 5-year cumulative HCC risk between 10–17% [13]. However, existing tools to predict the development of liver cancer are blunt and imprecise. Risks are reduced by antiviral therapy, suppression of VL to undetectable limits, and HBeAg seroconversion [14], and increased by a positive family history of liver cancer, coinfection with HCV,HDV, and excess alcohol. Excess mortality due to HCC is substantial (e.g. SMR 15.9 [15]). Framing HBV as a potent carcinogen could shape strategy approaches informed by other oncogenic threats, namely education, prevention, screening and risk reduction. For example, using public health approaches to tobacco smoking as a precedent, interventions aim to minimize any exposure, irrespective of the presence of end-organ disease in individual smokers.

## HBV as a syndemic challenge

HBV needs to be tackled alongside other physical and mental health challenges, and with insight into many other coexisting vulnerabilities linked to, for example, socioeconomic deprivation, homelessness, migration, racism, stigma and discrimination, sex work and substance misuse. Some of these groups face extreme marginalisation and harsh health inequities. A holistic view of HBV as part of a syndemic challenge highlights the need to focus on prevention and harm minimisation, targeted information, outreach testing with peer support, trauma-informed care, and linkage to services that can also provide care for diverse and complex health and social needs. The syndemic concept provides the basis for integrating HBV with existing clinical infrastructure, avoiding the vertical silo approach which is inefficient and inaccessible to many vulnerable groups. Decentralising services provides opportunities for HBV management to be combined into existing healthcare programmes (for example offering care through existing services for maternal and child health, sexually transmitted infections, migrant health), leading to better access and improved efficiencies as a result of shared use of resources and extended staff roles.

## Conclusion

The conventional paradigm of assessing HBV primarily as a liver disease (as enshrined in all clinical guidelines) is based on evidence of hepatic inflammation, fibrosis or cancer. This approach misses crucial opportunities to improve individual outcomes by intervening early to prevent complications. Taking a view of HBV as an infection, carcinogen or part of a syndemic challenge underpins strategies for wider screening, early intervention and robust public health interventions for prevention, and can reduce health inequities (Fig 1), providing foundations for progress towards elimination goals.
